# Supplementary material for: Molecular mechanism of parental H3/H4 recycling at a replication fork
Source: Nat Commun. 2024 Nov 2;15:9485. doi: 10.1038/s41467-024-53187-4 (PMC11531469; doi:10.1038/s41467-024-53187-4)
Supplement: Supplementary file 1 — Supplementary Information [file 41467_2024_53187_MOESM1_ESM.pdf]

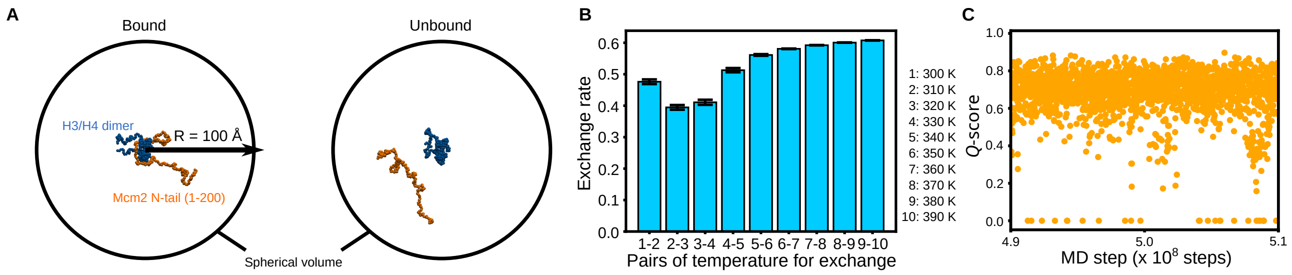

Supplementary Figure 1: Replica exchange molecular dynamics simulations of the Mcm2 N-tail and the H3/H4 dimer in a sphere. **(A)** Representative snapshots of the bound (left) and unbound (right) states. **(B)** Exchange rates between the replicas. The error bars represent the *mean* ± *standard deviation.* The standard deviations were calculated from three sets of simulations. **(C)** *Q*-scores between the Mcm2-7 N-tail and the H3/H4 dimer. The *Q*-score was defined as the ratio of residue-pair contacts at a particular time point to the natively formed contacts. We considered that a residue pair forms contact when these beads were within 1.2 times the distance in the native structure. Then, we defined the bound state as the state with $Q > 0$.

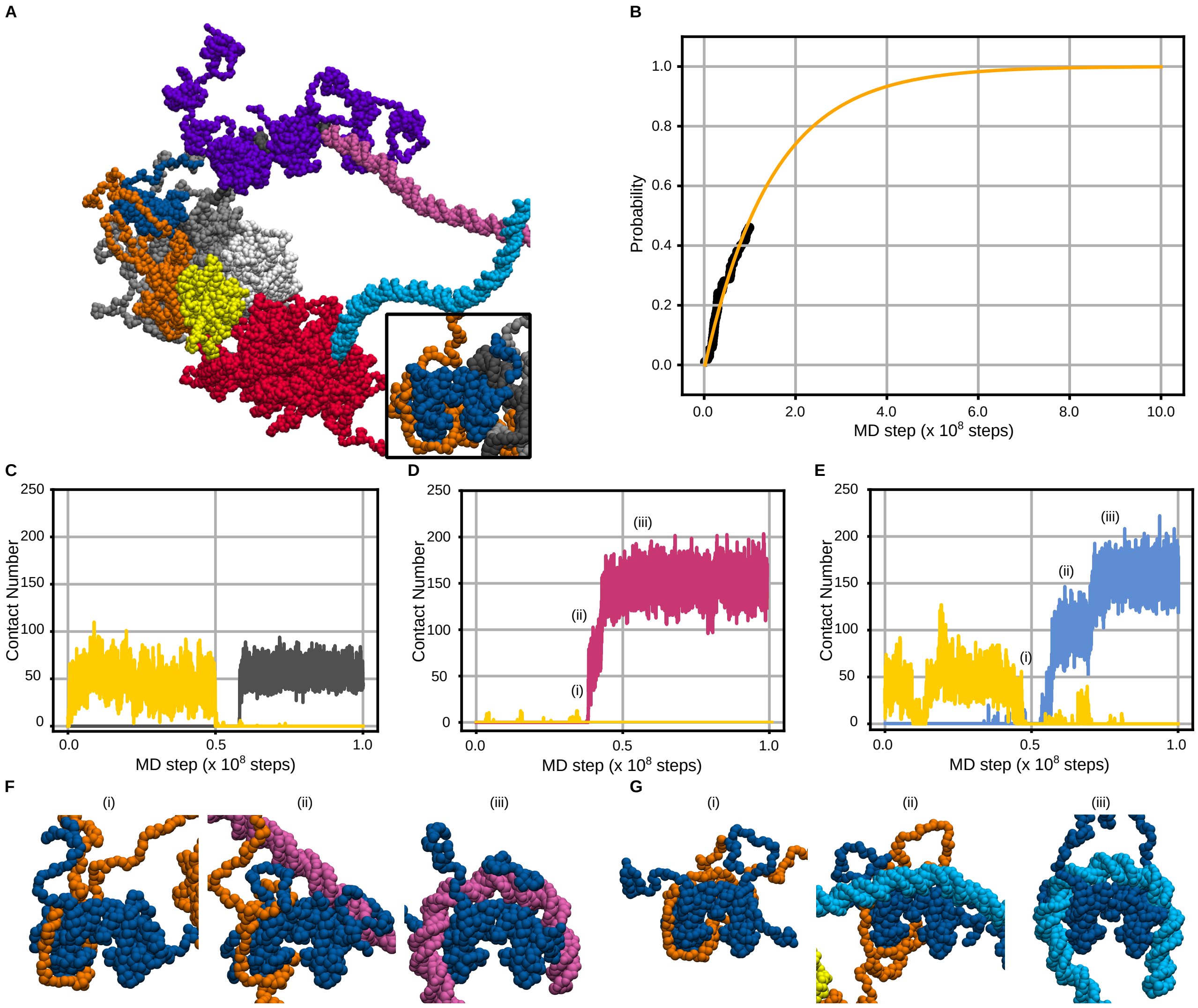

Supplementary Figure 2: The H3/H4 tetramer was deposited on the replicated strands by the Mcm2 N-tail in the simulations of the replicated-DNA-engaged replisome. **(A)** A representative snapshot of the H3/H4 tetramer deposited on the parental strand. **(B)** Cumulative probability of the H3/H4 tetramer being deposited on the parental, lagging, and leading strands. Data points (black) and a fitting curve (yellow) are shown (n = 46 trajectories). **(C-E)** Time trajectories of the number of residues in the H3/H4 tetramer contacting the parental strand (C, grey), the lagging strand (D, pink), the leading strand (E, cyan), and Cdc45 (C-E, yellow). **(F, G)** Representative snapshots of the H3/H4 tetramer recycled to the lagging (F) and leading (G) strands via the Cdc45-unmediated pathway.

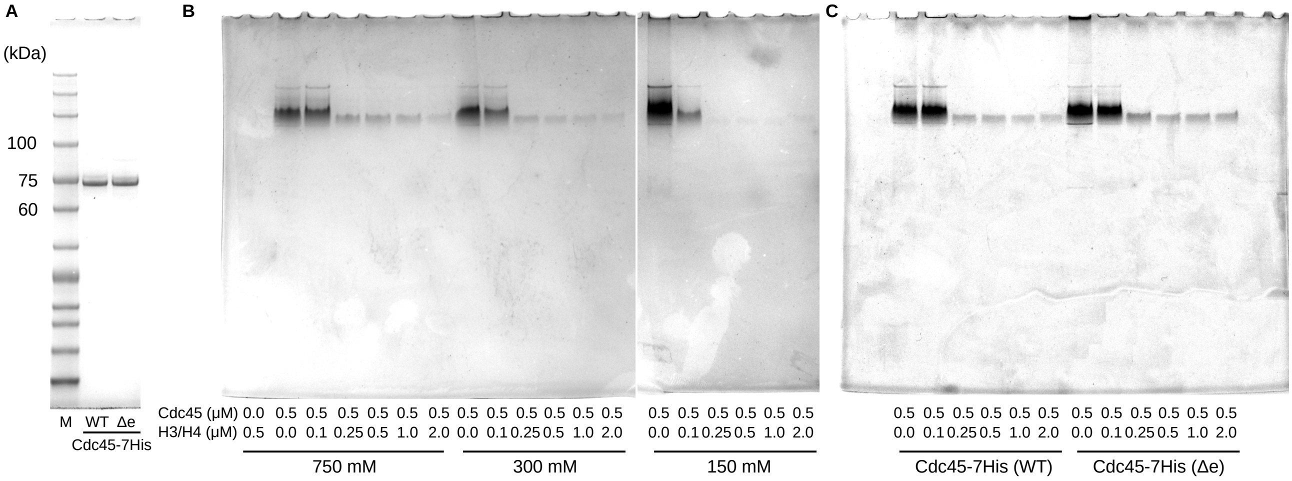

Supplementary Figure 3: Purification of Cdc45-7His and binding assays of Cdc45 and an H3/H4 tetramer. **(A)** The gel image of 7.5% SDS-PAGE of purified Cdc45-7His and Cdc45-7His (Δe). 'M' denotes a marker (Nacalai Tesque Inc.; 19593-25) **(B)** Gel images of 5–20% Native-PAGE of mixtures of Cdc45-7His and H3/H4 tetramer in 150, 300, 750 mM KCl (n = 3 independent experiments for each salt-condition). **(C)** A gel image of 5–20% Native-PAGE of mixtures of Cdc45-7His (or Cdc45-7His (Δe)) and H3/H4 tetramers in 300 mM KCl (n = 3 independent experiments).

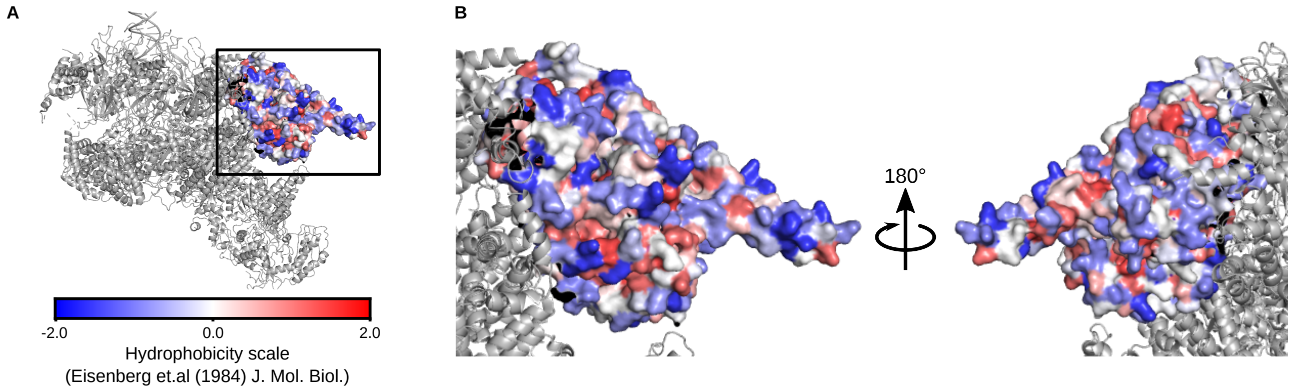

Supplementary Figure 4: Hydropathy-scale mapping on surface of Cdc45. (A) Structures (PDB ID: 6HV9[1]) and molecular surfaces of Cdc45 in a replisome colored according to hydrophobicity scale[2]. (B) Magnified views of the Cdc45 surfaces.

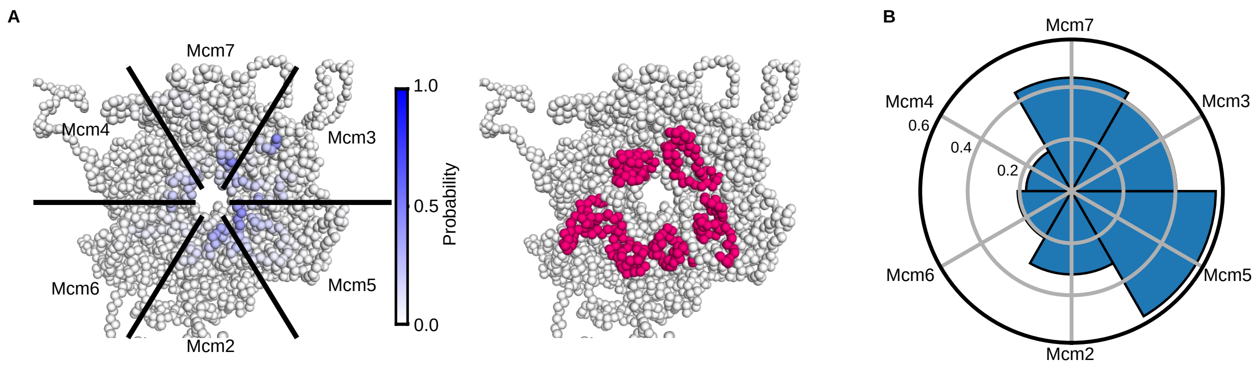

Supplementary Figure 5: Analysis upon contacts between Mcm2-7 and the ssDNA region of the lagging strand. **(A & B)** Structures of Mcm2-7 viewed from the N-terminal side. The residues in zinc-finger domains of Mcm subunits were colored pink (A). The shades of blue on the structure represent the probabilities of the residues in Mcm2-7 contacting the ssDNA region of the lagging strand (B). **(C)** The probabilities of the Mcm2-7 subunits contacting the ssDNA region of the lagging strand.

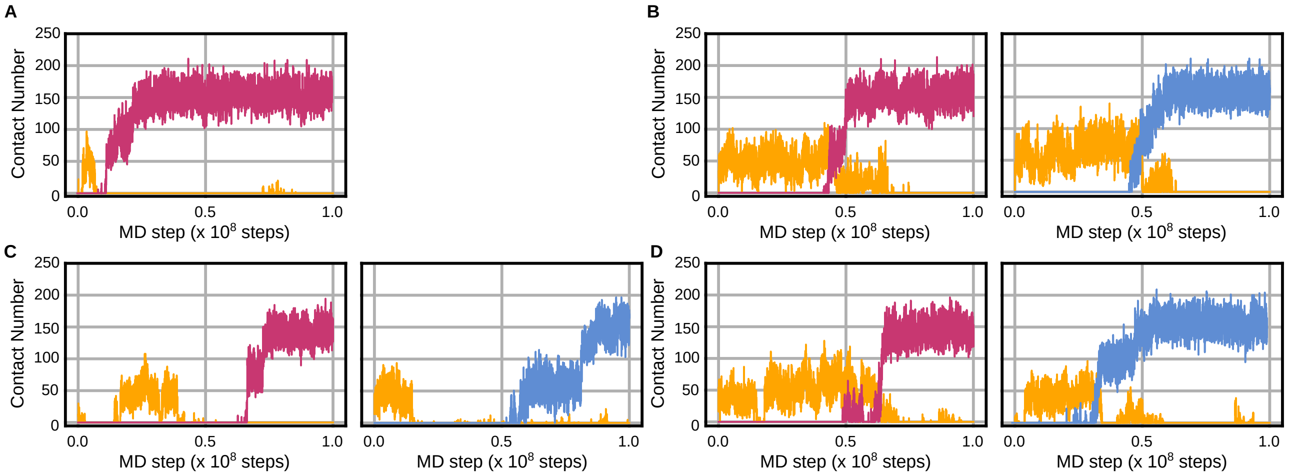

Supplementary Figure 6: Time trajectories of the number of residues in the H3/H4 tetramer contacting the lagging strand (pink), the leading strand (cyan), and Cdc45 (yellow) in the simulations of the replicated-DNA-engaged replisome with zero or one RPA. **(A, B)** The trajectories in which the H3/H4 tetramer was recycled via the Cdc45-unmediated (A) and mediated (B) pathway in the simulations with zero RPA. **(C, D)** The trajectories in which the H3/H4 tetramer was recycled via the Cdc45-unmediated (C) and mediated (D) pathway in the simulations with one RPA.

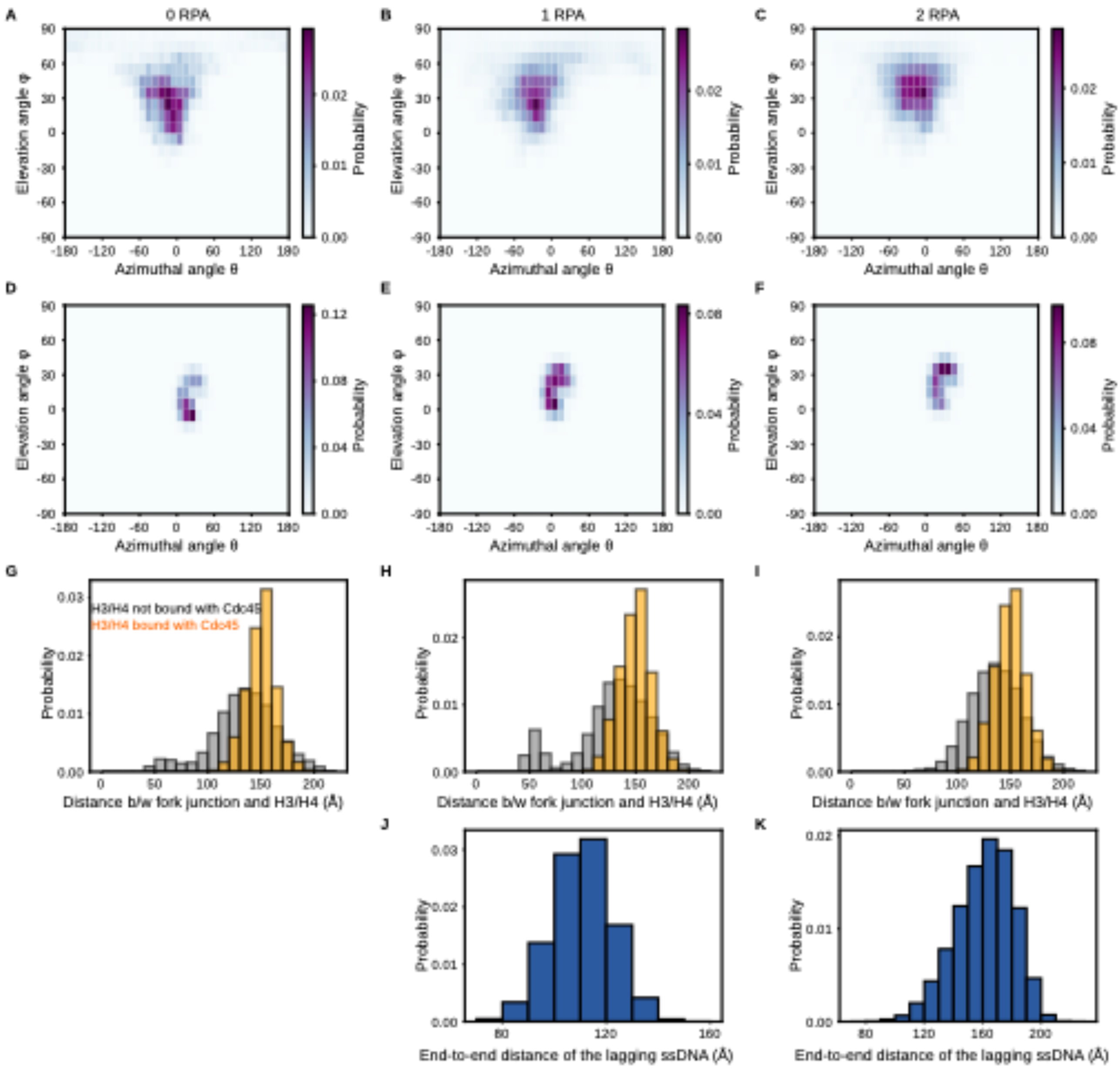

Supplementary Figure 7: Detailed analysis upon dynamics of H3/H4 tetramer in the simulations with 0-, 1-, and 2-RPA molecules. **(A-F)** 2D probability distributions of the H3/H4 tetramer not bound with Cdc45 (A, B, and C in the 0-, 1-, and 2-RPA case) and those of the H3/H4 tetramer bound with Cdc45 (D, E, and F in the 0-, 1-, and 2-RPA case) until recycling to the lagging strand. **(G-I)** 1D probability distributions of the distance between the H3/H4 tetramer bound with Cdc45 (gray) or not (orange) and the fork junction until recycling in the 0- (G), 1- (H), and 2-RPA (I) case. **(J, K)** 1D probability distributions of the end-to-end distance of the lagging ssDNA in the 1- (J) and 2-RPA (K) case.

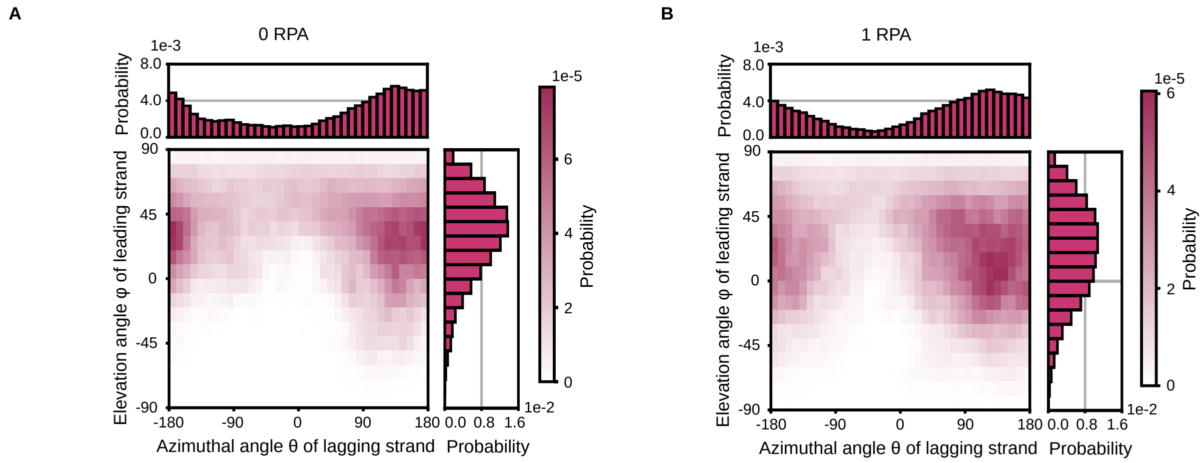

Supplementary Figure 8: 1D and 2D probability distributions of the lagging strand orientation calculated from the simulation with zero (A) and one (B) RPA molecule.

Supplementary Table 1: The DNA sequence used in experiments. The Autonomously Replicating Sequence 1 (ARS1) and the Widom 601 sequence are colored blue and red, respectively.

GCGCTAATACGACTCACTATAGGGGCGGCAATGTGAGTGTAGCACACGCATCAGCATTA<br>GCTTGCCGTTTTTACGCGCGTAACGGGTAACCAAGCCGTTCTTCCAAATACGATTAGGA<br>GAATCGTGGGGAAGGAGGTGTGGAGACAAATGGTGTAAAAGACTCTAACAAAATAGC<br>AAATTTCGTCAAAAATGCTAAGAAATAGGTTATTACTGAGTAGTATTTATTTAAGTATTGT<br>TTGTGCACTTGCCTGCAGGCCTTTTGAAAAGCAAGCATAAAAGATCTAAACATAAAATC<br>TGTAAAATAACAAGATGTAAAGATAATGCTAAATCATTTGGCTTTTTGATTGATTGTACA<br>GGAAAATATACATCGCAGGGGGTTGACCAGTGCACAAGGGAGGCGGGCTCATGCATCA<br>CGCAGTCTTTATCAGTAAATGAAGTACATCTAGTCCATGTGATAGTTTTAATTTGTAAGA<br>TCACGTGCTCCACATGGGTTCGTGGGCCCACATCGGATGTATATATCTGACACGTGCCT<br>GGAGACTAGGGAGTAATCCCCTTGGCGGTTAAAACGCGGGGGACAGCGCGTACGTGC<br>GTTTAAGCGGTGCTAGAGCTGTCTACGACCAATTGAGCGGCCTCGGCACCGGGATTCT<br>CGATAAGCTGTCGATTGCTCGCCCTACTGTCGTACACTCCGGGTAGCTCCACTCAGAAG<br>GTCCGTGAGTGGTAATAGGGTATTCTGTTTAATCCATTTCAGAAGCATTTGTGCACTGAT<br>CGCCCGCCACGGACCATTTCGTATGATTAACGCACTTATCTTAGTGCGACCAACTGGAT<br>CTCAATGGGGACTTCATTGGCCAGATTGGTCCCGGAAAGTAGTGTAAGGGGCCCGAGA<br>CTCTTCTTTGGATACGGGAAATACCTCAAGGAAGAGAATGCGTTAGTAGCTCGGCTACT<br>CCAGGAAGTAACGAACTTCGAGGCCCTTGAACCGGTTTGATTCTTCGAAACCAGTGCG<br>CGGATGGCTTTCATAATGATCATCTGGGGTCGACAAAAATGGCATTGGGCAAGAGGTGT<br>GCAATAATCCCATTAGCGTAGTGCAAGTAAGTCAGTAATAGCAATATTGTATACAGTCAC<br>GTTGGAACGAGAACGCGTGGCGTAATCATGGTGCGC

SUPPLEMENTARY REFERENCE

1. Goswami, P. *et al.* Structure of DNA-CMG-Pol epsilon elucidates the roles of the non-catalytic polymerase modules in the eukaryotic replisome. *Nat. Commun.* **9**, 5061 (2018).
2. Eisenberg, D., Schwarz, E., Komaromy, M. & Wall, R. Analysis of membrane and surface protein sequences with the hydrophobic moment plot. *J. Mol. Biol.* **179**, 125–142 (1984).
