# Supplementary material for: Molecular mechanism of parental H3/H4 recycling at a replication fork
Source: Nat Commun. 2024 Nov 2;15:9485. doi: 10.1038/s41467-024-53187-4 (PMC11531469; doi:10.1038/s41467-024-53187-4)
Supplement: Supplementary file 3 — Description of Additional Supplementary Files [file 41467_2024_53187_MOESM3_ESM.pdf]

Description of Additional Supplementary Files

Supplementary Movie 1: The representative trajectory in which the H3/H4 tetramer was deposited to the lagging strand via the Cdc45 mediated pathway.

Supplementary Movie 2: The representative trajectory in which the H3/H4 tetramer was deposited to the leading strand via the Cdc45 mediated pathway.

Supplementary Movie 3: The representative trajectory in which the H3/H4 tetramer was deposited to the lagging strand via the Cdc45 unmediated pathway.

Supplementary Movie 4: The representative trajectory in which the H3/H4 tetramer was deposited to the leading strand via the Cdc45 unmediated pathway.
