# Supplementary material for: Molecular mechanism of parental H3/H4 recycling at a replication fork
Source: Nat Commun. 2024 Nov 2;15:9485. doi: 10.1038/s41467-024-53187-4 (PMC11531469; doi:10.1038/s41467-024-53187-4)
Supplement: Supplementary file 8 — Reporting Summary [file 41467_2024_53187_MOESM8_ESM.pdf]

nature portfolio

Corresponding author(s): Tsuyoshi Terakawa

Last updated by author(s): Jul 8, 2024

Reporting Summary

Nature Portfolio wishes to improve the reproducibility of the work that we publish. This form provides structure for consistency and transparency in reporting. For further information on Nature Portfolio policies, see our Editorial Policies and the Editorial Policy Checklist.

Statistics

For all statistical analyses, confirm that the following items are present in the figure legend, table legend, main text, or Methods section.

n/a	Confirmed	
☐	☒	The exact sample size (*n*) for each experimental group/condition, given as a discrete number and unit of measurement
☐	☒	A statement on whether measurements were taken from distinct samples or whether the same sample was measured repeatedly
☐	☒	The statistical test(s) used AND whether they are one- or two-sided<br>*Only common tests should be described solely by name; describe more complex techniques in the Methods section.*
☒	☐	A description of all covariates tested
☒	☐	A description of any assumptions or corrections, such as tests of normality and adjustment for multiple comparisons
☐	☒	A full description of the statistical parameters including central tendency (e.g. means) or other basic estimates (e.g. regression coefficient) AND variation (e.g. standard deviation) or associated estimates of uncertainty (e.g. confidence intervals)
☐	☒	For null hypothesis testing, the test statistic (e.g. *F*, *t*, *r*) with confidence intervals, effect sizes, degrees of freedom and *P* value noted<br>*Give P values as exact values whenever suitable.*
☒	☐	For Bayesian analysis, information on the choice of priors and Markov chain Monte Carlo settings
☒	☐	For hierarchical and complex designs, identification of the appropriate level for tests and full reporting of outcomes
☒	☐	Estimates of effect sizes (e.g. Cohen's *d*, Pearson's *r*), indicating how they were calculated

Our web collection on statistics for biologists contains articles on many of the points above.

Software and code

Policy information about availability of computer code

Data collection	1. All the MD simulations were performed using the CafeMol 3.2 (https://www.cafemol.org).<br>2. Protein models were created using Modeller10.3<br>3. Figures were created using vmd1.9.3 (for movies and snapshot of protein and DNA structures), PyMol 2.4.0a0 (for ribbon diagram of protein and DNA strucrtures), iBright Firmware 1.8.1 (for gel images), ImageLab Touch 2.3 software (for gel images), matplotlib3.3.4 (for graphs), and Inkscape0.92.3 (for graphs)
Data analysis	We used the following programs for data analysis:<br>1. Fiji (https://imagej.net/software/fiji/)<br>2. Python 3.9.17 (https://www.python.org)<br>3. vmd1.9.3 (https://www.ks.uiuc.edu)

For manuscripts utilizing custom algorithms or software that are central to the research but not yet described in published literature, software must be made available to editors and reviewers. We strongly encourage code deposition in a community repository (e.g. GitHub). See the Nature Portfolio guidelines for submitting code & software for further information.

Data

Policy information about availability of data

All manuscripts must include a data availability statement. This statement should provide the following information, where applicable:

- Accession codes, unique identifiers, or web links for publicly available datasets
- A description of any restrictions on data availability
- For clinical datasets or third party data, please ensure that the statement adheres to our policy

The trajectory data generated in this study are available in the following database
BSM-ID BSM00050 (https://bsma.pdbj.org/entry/50)

Research involving human participants, their data, or biological material

Policy information about studies with human participants or human data. See also policy information about sex, gender (identity/presentation), and sexual orientation and race, ethnicity and racism.

Reporting on sex and gender	N/A
Reporting on race, ethnicity, or other socially relevant groupings	N/A
Population characteristics	N/A
Recruitment	N/A
Ethics oversight	N/A

Note that full information on the approval of the study protocol must also be provided in the manuscript.

Field-specific reporting

Please select the one below that is the best fit for your research. If you are not sure, read the appropriate sections before making your selection.

☒ Life sciences ☐ Behavioural & social sciences ☐ Ecological, evolutionary & environmental sciences

For a reference copy of the document with all sections, see nature.com/documents/nr-reporting-summary-flat.pdf

Life sciences study design

All studies must disclose on these points even when the disclosure is negative.

Sample size	We repeated biochemical experiments (native-PAGE experiments and replication assays) three times and confirmed that the standard deviation across the trials was minimal, indicating reliable consistency.For molecular dynamics simulations, we performed 50 independent runs for the 0-RPA and no-Pol epsilon case and 100 independent runs for the 1-RPA and 2-RPA cases with different random seeds, each comprising 10^8 steps. Sample sizes are determined to ensure that three or above trajectories show recycling to the lagging or leading strand in the pathway observed in the study.
Data exclusions	No data were excluded from analysis.
Replication	For biochemical experiments, we performed three times independent replicates were performed, confirming that the standard deviations of the results were sufficiently small. For molecular dynamics simulations, we performed 50-100 times independent simulations for each setup. In all cases, replication was successful.
Randomization	We prepared the reaction mixtures separately for replicates of biochemical experiments. All molecular dynamics simulations were performed with different random seeds to generate different states in a given phase space. In all the simulations, we introduced random forces through the use of the Langevin dynamics.
Blinding	Blinding is not applicable to biochemical experiments and molecular dynamics simulations as performed in this manuscript. Since it is not possible to trace the sample conditions exactly from the results (e.g., gel data), an analysis cannot be performed by a tester who does not know the sample condition. Samples are sufficiently randomized, and all conditions are identical except for those to be compared; therefore, the procedures without blinding are sufficient.

Reporting for specific materials, systems and methods

We require information from authors about some types of materials, experimental systems and methods used in many studies. Here, indicate whether each material, system or method listed is relevant to your study. If you are not sure if a list item applies to your research, read the appropriate section before selecting a response.

Materials & experimental systems

n/a	Involved in the study
☒	☐ Antibodies
☒	☐ Eukaryotic cell lines
☒	☐ Palaeontology and archaeology
☒	☐ Animals and other organisms
☒	☐ Clinical data
☒	☐ Dual use research of concern
☒	☐ Plants

Methods

n/a	Involved in the study
☒	☐ ChIP-seq
☒	☐ Flow cytometry
☒	☐ MRI-based neuroimaging

Plants

Seed stocks	*Report on the source of all seed stocks or other plant material used. If applicable, state the seed stock centre and catalogue number. If plant specimens were collected from the field, describe the collection location, date and sampling procedures.*
Novel plant genotypes	*Describe the methods by which all novel plant genotypes were produced. This includes those generated by transgenic approaches, gene editing, chemical/radiation-based mutagenesis and hybridization. For transgenic lines, describe the transformation method, the number of independent lines analyzed and the generation upon which experiments were performed. For gene-edited lines, describe the editor used, the endogenous sequence targeted for editing, the targeting guide RNA sequence (if applicable) and how the editor was applied.*
Authentication	*Describe any authentication procedures for each seed stock used or novel genotype generated. Describe any experiments used to assess the effect of a mutation and, where applicable, how potential secondary effects (e.g. second site T-DNA insertions, mosiacism, off-target gene editing) were examined.*
